# COVID-19: Targeting Proteases in Viral Invasion and Host Immune Response

**DOI:** 10.3389/fmolb.2020.00215

**Published:** 2020-10-09

**Authors:** Sanchit Seth, Jyotsna Batra, Srilakshmi Srinivasan

**Affiliations:** School of Biomedical Sciences, Faculty of Health, Translational Research Institute, Queensland University of Technology, Brisbane, QLD, Australia

**Keywords:** SARS-CoV-2, proteases, immune response, invasion, COVID-19

## Abstract

An acute respiratory disorder (COVID-19) that accelerated across the globe has been found to be caused by a novel strain of coronaviruses (SARS-CoV-2). The absence of a specific antiviral drug or vaccination has promoted the development of immediate therapeutic responses against SARS-CoV-2. As increased levels of plasma chemokines and, cytokines and an uncontrolled influx of inflammatory cells were observed in lethal cases, it was concluded that the severity of the infection corresponded with the imbalanced host immunity against the virus. Tracing back the knowledge acquired from SERS and MERS infections, clinical evidence suggested similar host immune reactions and host ACE2 receptor-derived invasion by SARS-CoV-2. Further studies revealed the integral role of proteases (TMPRSS2, cathepsins, plasmin, etc.) in viral entry and the immune system. This review aims to provide a brief review on the latest research progress in identifying the potential role of proteases in SARS-CoV-2 viral spread and infection and combines it with already known information on the role of different proteases in providing an immune response. It further proposes a multidisciplinary clinical approach to target proteases specifically, through a combinatorial administration of protease inhibitors. This predictive review may help in providing a perspective to gain deeper insights of the proteolytic web involved in SARS-CoV-2 viral invasion and host immune response.

## Introduction

Since early 2020, the world has been experiencing the uncontrollable spread of novel coronavirus (2019-nCOV) dependent acute respiratory illness, the outbreak of which was first reported in the city of Wuhan, Hubei, China in late 2019. As of May 11, 2020, there have been more than four million confirmed cases globally with over 280,000 deaths till date. The common clinical symptoms in patients include fever, cough, and fatigue, with gastrointestinal infection for a small group of the population (Wu F. et al., 2020). Next generation sequencing (NGS) revealed a genome sequence similarity between, and distinct genomic composition of, the causative agent of SARS-CoV and MERS-CoV, identified in 2002/03 and 2011 respectively, that caused global pandemics of Severe Acute Respiratory Syndrome (SARS) and Middle East Respiratory Syndrome (MERS) (Wu F. et al., 2020). Full-length genome sequences obtained from five patients suffering from an early stage of illness revealed a 79.6% sequence identity to SARS-CoV and 96% identicality at the whole-genome level to a bat coronavirus (Zhou et al., 2020). Hence, on February 11, 2020 the World Health Organisation (WHO) officially named the disease as coronavirus disease 19 (COVID-19) and the new corona virus as SARS-CoV-2. Virus infectivity tests showed angiotensin converting enzyme II (ACE2) receptor as the cellular entry receptor, which is the previously known cell receptor for SARS-CoV (Wu F. et al., 2020) for human infections. The genomic and clinical symptomatic identicality observed between SARS-CoV and SARS-CoV-2 allowed researchers to predict the host immune response and explore how SARS-CoV-2 may evade the host response. This review focuses on gathering recent clinical data on the host immune response and correlating these with our current knowledge about the viruses, providing updates on the application of host cell proteases as a potential therapeutic approach against COVID-19 viral replication, and discussing the severity of disease.

## SARS-CoV-2: Genomic Structure and Pathogenesis

Isolation of one strain of SARS-CoV-2 from a COVID-19 pneumonia patient from Wuhan seafood market revealed to be 29.9 kb (Wu F. et al., 2020) non-segmented positive-sense RNA β-coronavirus (Zhu et al., 2020). When compared to the RNA genome of SARS-CoV and MERS-CoV, 27.9 and 30.1 kb, respectively (de Wit et al., 2016), SARS-CoV-2 contains 6-11 open reading frames (ORFs). Two polyproteins, pp1a and pp1ab, and 16 non-structural proteins (NSP) are encoded by the first ORF (ORF 1a/b) which covers two-thirds of the viral RNA. The remaining viral genomes encode several accessory proteins and four essential structural proteins, including small envelope (E) protein, nucleocapsid (N) protein, spike (S) glycoprotein, and matrix (M) protein (Cui et al., 2019), involved in potential interference with the host innate immune response. Meta-transcriptomic sequencing on the same sample showed the genomic and phylogenetic similarity of SARS-CoV-2 to SARS-CoVs, particularly in the S-protein and receptor binding domain (RBD), revealing the capability of human transmission of COVID-19 (Wu A. et al., 2020). The protein level mutation observed in NSP2, NSP3, RBD, and spike protein indicated its role in severity of infection, differentiation mechanism, and spread of the disease (Angeletti et al., 2020).

The cross-species and human-to-human transmission of COVID-19 through the ACE2 cellular entry receptor was confirmed by Zhou et al. (2020). It involves the attachment of virion *S*-glycoprotein to the ACE2 receptor presented by human cells through the S1 sub-unit, leading to the virus-membrane fusion by the S2 sub-unit of the glycoprotein (Zhang et al., 2014). Upon fusion, the viral genomic RNA is released in the cytoplasm, translating the non-structural proteins to form replication-transcription complex (RTC) (Sawicki and Sawicki, 2005). Continuous replication of RTC leads to the synthesis of sub-genomic RNAs, encoding the structural and accessory proteins (Hussain et al., 2005). Newly formed genomic RNA envelope glycoproteins and nucleocapsid proteins and, interact with endoplasmic reticulum and golgi to form viral particle buds that finally fuse with the plasma membrane of the host cell to release the virus (Perrier et al., 2019).

As it is established that SARS-CoV-2 shares the same cellular entry receptor as SARS-CoV, the ACE2 binding efficacy of S-protein was observed to be 10–20 fold higher in SARS-CoV-2 in a recent study (Song et al., 2018). It was also observed that human cells expressing ACE2 but not human Dipeptidyl peptidase-4 (DPP4) or Aminopeptidase N (APN) were less susceptible to the human cell entry of SARS-CoV-2 (Letko et al., 2020). The priming of Spike (S) protein following the binding to the ACE2 receptor results in the viral- host cell fusion (Tortorici and Veesler, 2019). The cathepsin L-dependent viral glycoprotein activation via SARS-CoV S-protein cleavage at S1/S2 boundary under low pH conditions and involvement of transmembrane protease serine 2 (TMPRSS2) in triggering the cleavage of trimer S-protein (Simmons et al., 2005; Millet and Whittaker, 2015) opened new avenues to study the role and participation of different proteases in the endocytosis of SARS-CoV-2 into human cells as well as potential for drug targeting and vaccine development. The observed increase in binding efficiency of SARS-CoV-2 *S*-glycoprotein to the host receptor can be related to the codon mutations observed in the protein sequence, resulting in a plausible increase in the site-specific priming activity of proteases and cathepsins, leading to the highly contagious nature of SARS-CoV-2 as compared to SARS and MERS. Proteases belonging to the proprotein convertase family, including furin and furin-like serine proteases, were analyzed for their ubiquitous role in viral entry and spread. Although produced in the endoplasmic reticulum and role in the viral biosynthesis, these furin and furin-like proteases were found translocated through secretory pathways to access viral S-protein and promote viral entry to host cells (Seidah and Prat, 2012). A recent study presented results supporting the presence of a furin-like cleavage site in the spike protein of SARS-CoV-2, which was absent in the other beta coronaviruses (Coutard et al., 2020). It was previously presented that, compared to lower pathogenic forms of the influenza virus, highly pathogenic versions selectively possess a furin-like cleavage site, replaced by a single basic residue cleavage site present in the less pathogenic viruses (Sun et al., 2010; Kido et al., 2012). Another group of researchers correlated the presence of a furin site in the envelope protein to the elevated levels of plasminogen observed in severe COVID-19 patients (Ji et al., 2020). Further research focusing on site-specific binding studies could be an approach to reveal potential druggable targets involving different proteases and specific peptide inhibitors.

## SARS-CoV-2: Human Immune Response and Immunopathology

The preliminary host innate immune response initiates as soon as the viral particles (RNAs and viral proteins) enter the host cell. Pattern recognition receptors (PRRs), including Toll-like receptors (TLR)-3, TLR-7, TLR-8, and TLR-9 (Alexopoulou et al., 2001; Wu and Chen, 2014), endosomal RNA receptor, and the cytosolic RNA sensor (RIG-I/MDA5), recognizes the pathogen-associated molecular patterns (PAMPs) (viral proteins and nucleic acids), induces complex signaling to activate transcription factor nuclear factor-κB (NFκB) and interferon regulatory factor-3 (IRF-3), which leads to the production of type I Interferons (IFN-α/β) (Kawai and Akira, 2010) and the recruitment of a series of pro-inflammatory cytokines, acting as the first line of defense against virus invasion. The production of IFN-α/β further initiates the transcription of IFN-stimulated genes (ISGs) via the activation of the JAK-STAT pathway and formation of a complex with IRF9 (de Wit et al., 2016). Hence, a successful initiation of IFN-α/β response should lead to the suppression of viral replication at an early stage. While immune response plays a crucial role in combating an infection, it may also result in immunopathogenesis. Research derived from SARS infection cases correlated acute respiratory distress syndrome (ARDS) to the early activation of IFN-γ and IFNα and upregulation of proteins encoded by ISGs, pro-inflammatory cytokines, and chemokines, particularly IL-6, -8, CXC-chemokine ligand 10 (CXCL10), and CC-chemokine ligand 2 (CCL2) (Tang et al., 2005; Baas et al., 2006). Furthermore, in comparison to the survivors of SARS-CoV infections, low levels or an absence of spike-specific antibodies were observed in patients who succumbed to disease, suggesting the relatability of disease severity to the lack of an innate adaptive immune response switch (Cameron et al., 2007).

Depending on the duration and severity of the disease, varied host innate immune responses were observed from the limited information available on patients suffering from COVID-19. In one of the reports from Wuhan, increased levels of serum IL-6, neutrophils, and C-reactive protein with reduced total lymphocytes were observed in a study on 99 patients (Zhou et al., 2020). Another independent study of 41 patients presented similar results for patients admitted to the Intensive Care Unit (ICU) vs. non-ICU patients, correlating the increase of neutrophils and reduction of lymphocytes to disease severity and death (Wu F. et al., 2020). Furthermore, in COVID-19 patients, elevated levels of plasma chemokines and cytokines, including granulocyte colony-stimulating factor (G-CSF), macrophage colony-stimulating factor (MCSF), hepatocyte growth factor (HGF), IP-10, MCP-1, IFN-γ, TNF-α, MIP-1α and IL -1, -2, -4, -7, -10, -12, -13, and -17, were observed (Chen et al., 2020; Wang et al., 2020). These clinical observations directed toward the involvement of highly pro-inflammatory conditions, like what was seen during SARS-CoV and MERS-CoV infections, further suggest a similar cytokine storm-mediated severity of the disease. An anatomy report of a deceased patient suffering from COVID-19 showed a severe inflammatory response in the lower airway, causing lung injury (Guo et al., 2020), potentially caused by a “cytokine- storm.”

Based on the limited current data and similarity of COVID-19 to earlier CoV infections, it can be predicted that innate immune response plays an important protective or destructive role, depending on the progression of the disease. Sharing the same ancestor as SARS-CoV, there is a possibility that both structural and non-structural viral proteins of SARS-CoV-2 might interfere with the activation of type 1 IFN, resulting in the alteration of its production (Shahabi et al., 2020). Hence, active viral replication in the early stage could later result in hyperproduction of type 1 IFN, leading to a great influx of pro-inflammatory cytokines, including neutrophils and macrophages, causing complications like ARDS, arrythmia, shock, and fatal pneumonia (Wang et al., 2020). Being the key link between the innate and active immune response switch (Tang et al., 2008), in-depth research aiming at targeting IFNs could be an optimum approach to control the imbalance in the immune reactions occurring at the later stage of the infection.

## SARS-CoV-2: Human Proteases Involvement in Invasion and Immune Response

**FIGURE 1:**
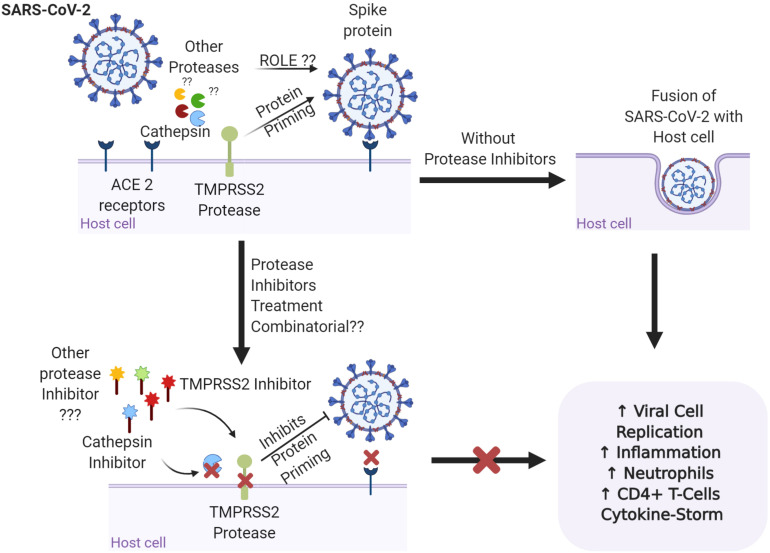
Representation of the binding of the spike protein on the surface of SARS-CoV-2 to the ACE2 receptors on the surface of the host cell. TMPRSS2 and cathepsin are involved in priming of the S glycoprotein and promoting viral invasion, leading to imbalance in the immune response and disease severity. Are other proteases involved in the proteolytic cascade of viral invasion? Targeting the proteases with specific protease inhibitors either alone or in combination may have better therapeutic potential for SARS-COV-2 infections. Figure prepared on biorender.com, professional science figure creating platform.

The role of proteases in SARS-CoV-related viral transmissibility and replication has long been under investigation. It has been presented previously that the entry of virus into the host cell was facilitated by the binding of the surface sub-unit, S1, of the S glycoprotein to the cell surface. Furthermore, the involvement of host proteases (Table 1) in priming the S glycoprotein at S1/S2 and S2’ sites has been confirmed by various researchers. A recent study revealed S-protein priming activity by host serine protease TMPRSS2, allowing the ACE2 receptor dependent viral and host cell membrane fusion via S2 subunit (Figure 1) (Matsuyama et al., 2010; Glowacka et al., 2011). Milewska et al. (2020) described the role of kallikrein-related peptidase 13 (KLK13) in specific cleaving of the S1/S2 region in Human coronavirus HKU1, directed toward the potential administration of protease inhibitors to inhibit viral entry. Following the similarity between SARS-CoV and SARS-CoV-2’s mode of entry by the ACE2 receptor, a recent study by Hoffmann et al. (2020) revealed the partial blockage of S-protein driven entry of SARS-CoV-2 by administering clinically proven serine protease inhibitor camostat mesylate. Further, full inhibition was observed when the camostat mesylate was coupled with Cathepsin B/L inhibitor E-64d, indicating alternate protein priming by endosomal cysteine proteases Cathepsin B/L (Hoffmann et al., 2020). These results were in concert with another study where SPINT2 gene-encoded protease inhibitor targeted TMPRSS2 and led to the restriction of cleavage-activation and viral growth for a range of influenza viruses (Straus et al., 2020). Furthermore, the role of KLK1 and KLK5 in influenza viral infection enhancement and its inhibition by Kallistatin was well established by Leu et al. (2015) and Magnen et al. (2017). Knowing the co-localization and involvement of TMPRSS2 in activating other serine proteases like, KLK4 and KLK14 (Reid et al., 2017), the recently presented results offers the potential to understand the proteolytic web cascade aiding SARS-COV-2 entry into the host cell, and the combinatorial inhibitor administration approach could be considered beneficial to restrict the viral entry.

**TABLE 1:** Summarizing potential host proteases contributing to viral entry and uncontrolled immune response against viral infection.

**Causative agent**	**Contributing host protease**	**Mechanism involved**	**References**
SARS-CoV	HAT	Spike protein priming, leading to ACE2 receptor dependent viral entry	Bertram et al., 2011
	TTSPs		
	Factor Xa		
	TMPRSS11a		
SARS-CoV-2	TMPRSS2		
	Cathepsin B/L		
HKU1	KLK13	Involved in assisting human coronavirus entry by specific cleaving at S1/S2 site	Milewska et al., 2020
Influenza Virus	KLK1	Involved in cleavage of hemagglutinin, enhancing the viral production	Leu et al., 2015
	KLK5		Magnen et al., 2017
Neutrophils	Cathepsin C	Activation of several pro-inflammatory serine proteases	Méthot et al., 2007
	Proteinase 3	Overexpression leads to uncontrolled degradation of extracellular matrix and inflammatory responses	van der Geld et al., 2001
	Cathepsin G		Mambole et al., 2008
	Neutrophil Elastase	Affects the levels of growth factor α and stimulates mucus secretion.	Nakamura et al., 1992; Roghanian and Sallenave, 2008
Mast Cells	Tryptase, Chymase	Due to absence of protease inhibitors in lungs, uncontrolled elevated expression of granzymes lead to degradation of extracellular matrix and induce the induction of pro-inflammatory cytokines.	Pejler et al., 2007
NK cells	Granzymes A, B, H, K and M		Morice et al., 2007
T cells			Zhu et al., 2009; Jahrsdörfer et al., 2010
B cells			Hagn et al., 2009

**FIGURE 2:**
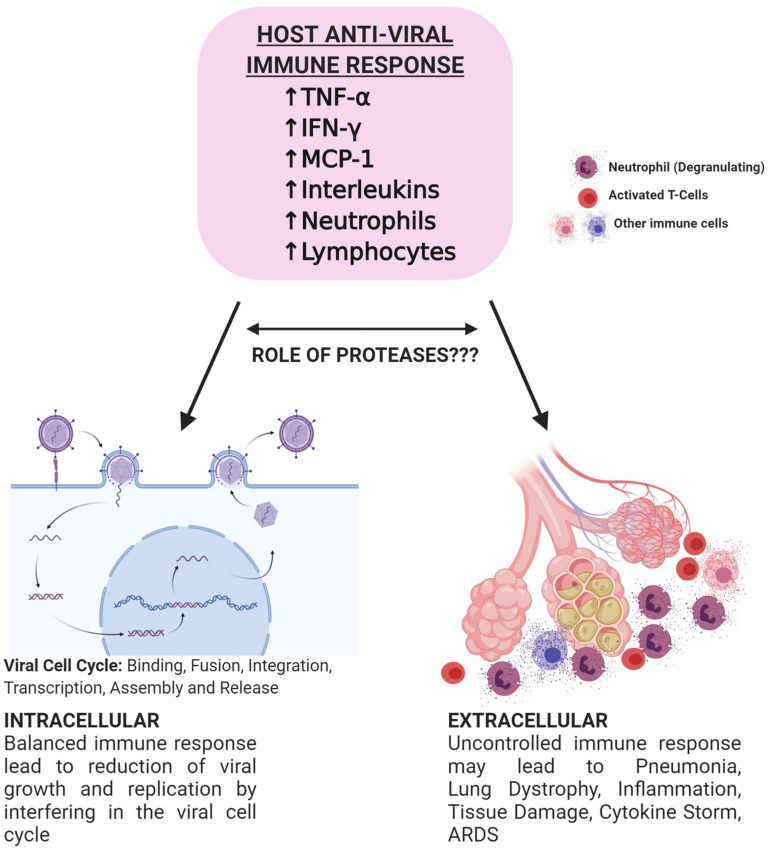
Schematic representation of potential bi-directional innate immune response against viral infections. Successful initiation of IFN-α/β related response leads to the suppression of viral infection at the early stage. SARS-CoV-2 hinders type 1 IFN related response and leads to the active replication of the virus. Increased viral load later causes hyperactivation of the immune system and may lead to lymphopenia, neutrophilia, and thrombocytopenia. Immune cells secrete proteases that play roles in the alteration of immune response. Targeting proteases may aid in controlled immunity and decrease the severity of the disease. Figure prepared on biorender.com, professional science figure creating platform.

Proteases also play a crucial role in presenting an immune response against the infection. Immune cells express a variety of serine proteases like neutrophil elastase, mast cell tryptase, and thrombin (Table 1) (Heutinck et al., 2010). Following the pattern of immune-pathogenic reactions and cytokine storms observed in fatal cases, a vivid correlation between an imbalanced immune response and disease severity led to the prospect of targeting proteases to eventually control the higher threshold of immune response (Figure 2). A recent study has revealed the association between human serine proteases trypsin, thrombin, tryptase, and elastase with increased expression of MCP-1 (Wang et al., 2007). Inhibition of these proteases resulted in the inhibition of MCP-1 secretion via inactivation of various protease-activated receptors (PARs), leading to the abolishment of its chemotactic activity (Wang et al., 2007). Furthermore, neutrophils respond to viral infections by activation of neutrophil elastase, Cathepsin G, and proteinase 3, playing roles both intracellularly and extracellularly (Méthot et al., 2007; Heutinck et al., 2010). However, activity of neutrophil proteases hinders the viral cell cycle and growth intracellularly and the presence of a high number of neutrophils at the inflammation site could correspond to imbalanced protease activity. This further leads to various inflammatory disorders, tissue damage, lung dystrophy, ARDS, and potentially death (Heutinck et al., 2010). Heutinck et al. (2010) showed significant correlation of neutrophil-derived and overexpressed KLK1 with the inflammation of the respiratory tract (Magnen et al., 2017). Relating to the similar clinical diagnosis for COVID-19, it can be deduced that serine proteases play an integral role in both viral invasion and adverse host immune response, further stimulating the pathway for more comprehensive investigations in relation to the specific activity of these proteases and their inhibitors to combat death-causing severity of the disease.

## COVID-19 Focused Treatments

Due to the lack of antiviral therapies against the novel virus and the rapid spread of the outbreak globally, research groups are focusing on treatments using the knowledge previously gathered from SARS and MERS coronaviruses. The current treatments are broadly focused on alleviating symptoms and offering respiratory support using oxygen therapy, ventilators, etc. Antiviral drugs, including ganciclovir, ribavirin, peramivir, and methylprednisolone, previously clinically practiced for influenza virus, failed to show response against SARS-CoV-2 (Li et al., 2020; Wang et al., 2020). Currently, patients diagnosed with COVID-19 are given a combination of nucleotide prodrug remdesivir and repurposed drug chloroquine, earlier used to treat malaria (Savarino et al., 2003; Li et al., 2020). While chloroquine inhibits the pH-dependent viral replication steps and acts as the immunomodulator by suppressing the activation and release of IL-6 and TNF-α (Savarino et al., 2003; Golden et al., 2015), remdesivir is believed to interfere with the NSP12 polymerase (Agostini et al., 2018), intravenous administration of which resulted in the decline of viral load detected on a nasopharyngeal swab of a first case of COVID-19 in Washington, United States (Ko et al., 2020). Administration of convalescent plasma derived from a COVID19-recovered patient with high SARS-CoV-2 specific antibody and neutralizing titre to five critically ill patients led to the improvement of their clinical symptoms (Shen et al., 2020). As these results were not obtained in controlled conditions, further clinical trials and studies would be required to understand the antiviral viral effect of the drugs and therapy on the immune system in bringing down the viral load.

Gathering the existing knowledge regarding vaccine development in SARS and MERS infection, many research groups have initiated the development of vaccines against SARS-CoV-2 considering different vaccine platforms like nucleotide-based, subunit-based, inactivated virus, and live attenuated virus-based platforms (Prompetchara et al., 2020). Full-length S1 proteins containing RBD can be considered as a potential antigen target, as neutralizing bodies against S1 can hinder host cell attachment and infection (Du et al., 2009; Al-Amri et al., 2017). As, the vaccine development would be the final prophylactic approach against SARS-CoV-2, it is time consuming and requires substantial research on issues such as immunization route, scalability, immune protection, production, and target product profile (Prompetchara et al., 2020). In contrast, targeting key proteases (viral and/or host) involved in viral entry and proliferation can be considered as an effective approach to alleviate the epidemic. Recent research has confirmed a significant decrease of SARS-CoV-2 viral load in Korean patients diagnosed with COVID-19 (Lim et al., 2020) when treated with protease inhibitor lopinavir and ritonavir, earlier used to treat human immunodeficiency virus (HIV) (Cvetkovic and Goa, 2003). Further studies targeting other host proteases may include cathepsins, granzymes, serine proteases, and proteases derived from various immune cells, responsible for viral entry, propagation, and altered innate and adaptive immune response.

Collecting our current understanding on role of proteases, it is suggested that a two-armed clinical approach, focusing on the combinatorial inhibition of host/virus protease dependent viral entry and controlled immune response, could be applied for a more robust response against SARS-CoV-2. Researchers have already commenced investigating the NSP5 main protease (M^*pro*^, 3C) as a potential drug target because of its involvement in processing the proteins coded from viral RNA (Zhang et al., 2020). An important aspect to consider while manipulating the host protease activity would be the specificity of the drugs against the targeted protease. As these proteases might be involved in other biological processes, non-specific inhibition might lead to impairment of the regular physiological functioning of the host.

## Conclusion

The COVID-19 epidemic not only affected the health of patients suffering from the disease but also left the entire world in deep financial and psychological distress. Although no promising treatment or vaccine has yet been discovered, research groups have been able to understand the genome of the novel virus, aiding them to develop vaccines and drugs against the therapeutic targets. Utilizing the host translational machinery for viral growth and propagation, SARS-CoV-2 entry to host cell is driven by the S-protein by engaging ACE2 and TMPRSS2 cell receptors. The activation of these receptors depends on the activity of various host proteases. In addition, to assist the functioning of cell receptors, multiple proteases are involved in host immune response against viral invasion and immunopathology related to imbalanced immune activation. This review, in relation to the prior knowledge on SARS-CoV and MERS-CoV, suggests a multidisciplinary approach, targeting host proteases against the cellular entry of coronaviruses through *S*-glycoprotein and host immune response against SARS-CoV2. It is of interest to know that mutations observed in the spike protein of SARS-CoV-2 might lead to high affinity binding to the ACE2 host receptor in the presence of cathepsin and serine protease TMPRSS2 when compared to SARS-CoV host cell receptor attachment. Based on the understanding of the host immune response against viral infection, it could be brought into light that the imbalanced immune cells’ activity can lead to the self-destruction of the host, resulting in a high severity of COVID-19. The significant role played by serine protease TMPRSS2 and other proteases, like cathepsins, plasmin, and KLKs, in priming the S-protein and promoting viral-host cell fusion has opened new avenues to study the role of various proteases in different cellular factors involved in SARS-CoV-2 invasion. Furthermore, unraveling the proteolytic web involved in the host immune response might provide insights into disease severity and reveal therapeutic targets. It is believed that this knowledge may aid in overcoming the hindrance encountered in targeting the viral sub-units of constantly evolving strains of SARS-CoV-2 and aid to develop quicker and more effective responses to extreme outcomes of the ongoing pandemic.

## Author Contributions

SrS presented the concept of review and edited it. SaS wrote the manuscript. JB contributed to the editing of the manuscript. All the authors contributed to the article and approved the submitted version.

## Conflict of Interest

The authors declare that the research was conducted in the absence of any commercial or financial relationships that could be construed as a potential conflict of interest.
